# Management of Hypopituitarism

**DOI:** 10.3390/jcm8122153

**Published:** 2019-12-05

**Authors:** Krystallenia I. Alexandraki, Ashley B. Grossman

**Affiliations:** 1 Endocrine Unit, 1st Department of Propaedeutic Medicine, School of Medicine, National and Kapodistrian University of Athens, 115 27 Athens, Greece; alexandrakik@gmail.com; 2 Department of Endocrinology, Oxford Centre for Diabetes, Endocrinology and Metabolism, Churchill Hospital, University of Oxford, Oxford OX3 7LE, UK; 3 Centre for Endocrinology, Barts and the London School of Medicine, London EC1M 6BQ, UK

**Keywords:** hypopituitarism, pituitary insufficiency, growth hormone deficiency, secondary hypothyroidism, pituitary apoplexy, adrenal crisis, pituitary metastases, secondary hypothyroidism, secondary hypogonadism, diabetes insipidus, secondary adrenal insufficiency

## Abstract

Hypopituitarism includes all clinical conditions that result in partial or complete failure of the anterior and posterior lobe of the pituitary gland’s ability to secrete hormones. The aim of management is usually to replace the target-hormone of hypothalamo-pituitary-endocrine gland axis with the exceptions of secondary hypogonadism when fertility is required, and growth hormone deficiency (GHD), and to safely minimise both symptoms and clinical signs. Adrenocorticotropic hormone deficiency replacement is best performed with the immediate-release oral glucocorticoid hydrocortisone (HC) in 2–3 divided doses. However, novel once-daily modified-release HC targets a more physiological exposure of glucocorticoids. GHD is treated currently with daily subcutaneous GH, but current research is focusing on the development of once-weekly administration of recombinant GH. Hypogonadism is targeted with testosterone replacement in men and on estrogen replacement therapy in women; when fertility is wanted, replacement targets secondary or tertiary levels of hormonal settings. Thyroid-stimulating hormone replacement therapy follows the rules of primary thyroid gland failure with L-thyroxine replacement. Central diabetes insipidus is nowadays replaced by desmopressin. Certain clinical scenarios may have to be promptly managed to avoid short-term or long-term sequelae such as pregnancy in patients with hypopituitarism, pituitary apoplexy, adrenal crisis, and pituitary metastases.

## 1. Introduction

Hypopituitarism or pituitary insufficiency includes all clinical conditions that result in partial or complete failure of the anterior and rarely of the posterior lobe of the pituitary gland to secrete hormones. Hypopituitarism may be the result either of pituitary or hypothalamic dysfunction, the former interfering with pituitary hormone secretion (secondary dysfunction) and the latter with hypothalamic pituitary-releasing hormone secretion (tertiary dysfunction). A number of congenital or acquired, inherited or sporadic clinical entities may result in isolated deficiency (IHD) or multiple pituitary hormone deficiency (MPHD). Novel mutations are now recognised to be responsible for many patients with congenital hypopituitarism, although their presentation may be delayed. Hence, hypopituitarism includes central or secondary adrenal insufficiency (SAI) caused by adrenocorticotropic hormone (ACTH) deficiency, secondary hypothyroidism (SHT) caused by thyroid-stimulating hormone (TSH) deficiency, secondary hypogonadism (SHG) caused by deficiency of the gonadotroph hormones (Luteinising (LH) and Follicle Stimulating Hormones (FSH)), growth hormone deficiency (GHD), and central diabetes insipidus (CDI) caused by antidiuretic hormone (ADH or arginine vasopressin, AVP) deficiency [1].

The absent or insufficient replacement of pituitary hormones may not be compatible with life, particularly in the context of ACTH deficiency. Moreover, hypopituitarism *per se* has been associated with an increase in both morbidity and mortality. Patients with craniopharyngiomas in particular have a higher risk when compared to patients with other causes of hypopituitarism, with or without GH replacement [2,3]. On the other hand, a Swedish study showed that women with hypopituitarism have a relatively higher risk of diabetes mellitus (DM) type 2, myocardial infarction, cerebral infarction and fractures, compared with hypopituitary men [4]. Finally, pituitary apoplexy (PitAp) or adrenal crisis (AC) are characteristically life-threatening clinical entities [5,6].

These risks have mandated the need for evidence-based guideline for hormonal replacement in hypopituitary adults, either of isolated or combined hormonal insufficiency, and *Endocrine Society Clinical Practice Guidelines* have been recently issued for the management of hypopituitarism but also for each target-hormone deficiency either at a primary or a secondary level of hypothalamo-pituitary target endocrine gland axis [7,8,9,10,11,12]. Significantly, many novel research studies do not focus on the hormonal replacement *per se*, but on ways of administration and delivery of the drug in order to achieve a better compliance from the patients’ point of view, but also to most closely parallel the physiological circadian secretion of the deficient hormone, and thus to optimise quality of life (QoL).

## 2. Aetiology

A number of different aetiologies may result in reduced pituitary reserves, as summarised in Table 1. Generally, the usual sequential pattern for hormonal deficiencies starts from the loss of GH, followed by the gonadotropins, then TSH and ACTH. There are several exceptions to this order [7], such as the case of hypophysitis in which, independently of the cause, especially ACTH and TSH deficiencies are frequently the presenting deficits [13]. Hypophysitis, as an inflammation of the pituitary, may be caused by different triggers. It may be primary, or secondary to sella and parasellar lesions, systemic diseases, or drugs (recently the main cause is the use of immune checkpoint inhibitors, ICI) [14]. ICIs are used in the management of a number of solid and haematological malignancies and are monoclonal antibodies targeting immune checkpoints such as the cytotoxic T-lymphocyte antigen-4 (CTLA-4)—CD28 and the programmed cell-death-1 (PD-1)— PD-1 ligand 1 (PD-L1) [15]. They cause a T-lymphocyte activation and proliferation causing a restoration of anti-tumour immunity, reversing immune escape or evasion and promoting tumour cell death. Their disadvantages are the immune-related adverse events (AEs) that, in the endocrine glands, are mostly represented by hypophysitis and consequent pituitary hormonal deficiencies that are usually permanent [13]. However, other induced endocrine deficiencies include thyroiditis and a form of DM type 1.

Non-functioning pituitary adenomas (NFPAs) have been extensively studied, resulting in at least one pituitary deficiency due to either a mass effect from the compression of normal pituitary or/and functional abnormalities which may distort or compress the pituitary stalk. Hypogonadism may result either from compression of gonadotroph cells or from the stalk-compression-induced hyperprolactinaemia seen with NFPA: in the latter case, hyperprolactinaemia results from the inability of dopamine to be delivered to lactotroph cells and hence a loss of its inhibitory control [17]. Radiotherapy is occasionally used for residual or recurrent pituitary adenomas post-surgery. Moreover, it has also been utilised in the management of parasellar lesions (craniopharyngiomas, meningiomas, germinomas), other brain tumours, brain metastases and lymphomas. Pituitary insufficiency represents a well-documented late complication of radiotherapy, usually occurring several years after the radiotherapy [14]. Somatotroph cells seem to be the most vulnerable to damage followed by gonadotroph, thyrotroph and corticotroph cells [17]; although the major locus of damage may be vasculitis in the hypothalamus, the posterior pituitary gland is less sensitive to radiation injury [14]. Monitoring for such effects should be initiated not more than one year after radiotherapy by clinical evaluation and pituitary hormone assessment. Advanced radiation technologies such as stereotactic radiosurgery, cyber knife, fractionated stereotactic radiotherapy and proton beam therapy have been considered to be less harmful for healthy tissues [14], but this may not actually be the case. Empty sella may be secondary, commonly associated with partial or complete hypopituitarism, with or without hyperprolactinaemia or primary, usually with normal pituitary function [14].

## 3. Management of Hypopituitarism

The management of hypopituitarism as IHD or MPHD has recently been discussed as a *Clinical Practice Guideline* from the Endocrine Society [7]. The aim of hypopituitarism management is usually to replace the target-hormone of the disturbed hypothalamo-pituitary-endocrine gland axis. However, there are exceptions to this rule, such as GHD and SHG when fertility is the primary concern. Moreover, while hypopituitarism is usually permanent it may be also transient in cases of compression, inflammation (hypophysitis), surgery or functional suppression. The aim of hormone replacement is to minimise both symptoms and clinical signs of specific hormone deficiencies with safety, minimise inconvenience, and to maximise QoL.

### 3.1. Adrenocorticotropic Hormone (ACTH) Deficiency Replacement Treatment

The endogenous secretion of glucocorticoids (GCs) follows a 24-h circadian rhythm, with a morning peak at the point of sleep-to-waking and a nadir at midnight. This circadian rhythm parallels the activity of a primary circadian clock sited in the suprachiasmatic nucleus of the hypothalamus, this synchronising secondary circadian rhythms in other brain areas and in peripheral centers such as the adrenals, and indeed in all cells [28]. For this reason, in patients with SAI the cortisol rhythm may retain some residual function as opposed to primary AI (PAI), where the cyclic secretion is completely lost [29]. This latter fact, along with the fact that the physiological daily production of cortisol may be lower than previously believed [30], can readily result in overtreatment of patients with AI unless great care is taken. The GC most widely used for cortisol replacement therapy worldwide is immediate-release oral GC hydrocortisone (HC) in daily divided doses. The current recommendations suggest total daily replacement doses for HC of 10—12 mg/m^2^ body surface area (BSA) [7]; in adults, the suggested HC dose is 15–20 mg as a total daily dose with the highest HC dose (half or two-thirds of the total daily dose) given immediately on waking (morning) and the other 2–3 (and rarely 4) doses given later at lunch and then late afternoon (or an additional early afternoon), respectively [7]. In general, dose adjustments depend on clinical status, patient preference, and co-morbidities: a HC ‘day-curve’ (HCDC) may have some merit in selected cases to establish the actual serum cortisol levels. However, even with these regimes minimising the overall daily dose and dividing the doses, patients still report problems with replacement therapy in both PAI and SAI, complaining of an impaired health-related QoL, specifically of fatigue [7,8,31,32]. The last dose should be taken in at least 6 h away from bedtime to avoid sleep disorders or metabolic side-effects [8]. Increased HC doses may need to be considered when enzyme-inducing drugs are co-administered since they increase GCs metabolism. Urinary free cortisol (UFC) measurement has the disadvantage in being used to adjust the HC dose as saturation of corticosteroid-binding globulin (CBG) following oral HC results in supraphysiological UFC excretion [1,33], resulting in a wide inter- and intra-variability [1,7,34]. As noted above, a HCDC which measures serum cortisol levels throughout the day may be helpful in dose adjustments with targets to avoid excess peaks (> 1000 nmol/l) or troughs (< 100 nmol/l) levels [35]. Measuring a salivary cortisol day curve may prove to represent a more reliable and convenient way of titrating doses and timing in some cases [1,36,37].

In the context of longer-acting GCs, the recent guideline reports that dexamethasone should be avoided, whereas prednisolone can be given in patients with reduced compliance or the non-availability of HC, and may be considerably cheaper [7]. Prednisolone is given in the morning as a single-doses ranging 3–5 mg. Its advantage is represented by the unique administration and its prolonged action 12–36 h, 4 to 5 times higher compared to 6–10 h for HC. Its disadvantage is that it cannot be routinely measured rendering dose adjustment problematic. Cortisone acetate is another immediate-release oral GC that has to be converted to HC by the enzyme 11β-hydroxysteroid dehydrogenase (11β-HSD1) type 1, and possesses GC activity 0.8 times that of HC [38,39], but is of limited use in Europe. However, in patients with documented 11β-HSD1 deficiency only HC is recommended [40]. A retrospective study in PAI and SAI patients reported a similar cardiometabolic risk between patients treated by HC (mean dose 20.5 mg daily) and prednisolone (mean dose 3.7 mg daily). However, satisfaction scores were higher in the prednisolone group, suggesting that the once-daily dose is more convenient than HC three-times daily [41]. When a patient is already taking GC replacement, biochemical testing for dose titration should be performed at least 18–24 h after the HC dose or longer for synthetic GCs, such as > 36 h for prednisolone. However, the recent guideline is in favour of using clinical features in the primary assessment of under-treatment or overtreatment than the HCDC previously used and UFC measurement [1,7,34].

Once-daily modified-release HC (MRHC) formulation (Plenadren) has been recently introduced and it is composed ofan immediate-release coating combined with an extended-release core. This formulation aims to model a more physiological cortisol secretion profile without troughs and peaks but with higher concentrations during the morning in the first 4 h of its administration and a period without cortisol-exposure at night, resulting in decreased 24-h exposure to cortisol [42]. Plenadrenresults in less recurrent infections and a better metabolic profile along with a less impaired QoL [38,43]. Another MRHC (Chronocort) administered twice-daily mimics also the physiological cortisol circadian rhythm [44,45,46,47]. An oral immediate-release granule formulation of HC (Alkindi) has also been developed for paediatric use, with the advantage of available doses of 0.5 mg, 1 mg, 2 mg and 5 mg [48] (Table 2).

In cases of post-surgical or other presumed transient hypopituitarism, SAI replacement with an MRHC is not recommended for the practical reasons that there are no commercially available low or intermediate dose MRHC formulations; a switch to MRHC may be tried when SAI lasts more than 12 months [38].

The use of a continuous subcutaneous HC infusion (CSHI) has been reported in patients with PAI, demonstrating a more physiological cortisol circadian rhythm, a normalisation of morning ACTH and restoration of nocturnal serum cortisol levels, along with a better QoL profile compared to conventional HC doses [1,49]. However, the use of CSHI is an expensive and invasive technique, but it may be an option in particular patients with difficulty in swallowing or with allergies to currently used GCs [38].

It is relevant to especially emphasise the use of replacement in patients with ICIs as their therapeutic indications are continuously increasing. As mentioned above, hypophysitis related to ICI’s early affects ACTH secretion, and this needs to be promptly recognised and treated to avoid an AC. Besides hypopituitarism, pituitary inflammation may cause compression of sella and parasellar structures and high-dose GCs treatment may be needed not only as replacement but also as anti-inflammatory therapy, although the rationale for this is not strong and they are rarely necessary. Pituitary surgery may be needed in patients resistant to GCs. There may be permanent hypopituitarism due to the pituitary fibrosis and atrophy that develop following the inflammation [13,15].

In a large adult hypopituitary patients’ study from KIMS (Pfizer International Metabolic Database) on data in patients with untreated GHD receiving GC replacement, it was shown that in 2737 hypopituitary ACTH-sufficient (987) compared to ACTH-insufficient (1750) patients have a similar QoL [50]. Similarly, after one year of GH replacement, the improvement in QoL did not differ between ACTH-sufficient and -insufficient patients, with no association seen between HC equivalent dose (HCeq) and QoL improvement. GC daily doses and QoL were significantly associated, with patients receiving low GC doses showing a better outcome compared to patients receiving HCeq doses of 25 mg or more, particularly in the context of tiredness, tenseness and social isolation. These data are retrospective and they have to be interpreted with caution, since the results could be due either to supraphysiological GC exposure *per se* or to the fact that physicians gave increased GC doses in order to address unmet symptoms (such us unexplained fatigue).

Importantly, it is crucial to consider the appropriate patients’ education regarding stress-dose and emergency GC administration, with the emergency card/bracelet/necklace regarding AI and an emergency kit containing injectable high-dose GC [7,8,34]. When an AC due to SAI is suspected, parenteral injection of 50–100 mg HC should be given immediately, either intramuscularly (IM) or subcutaneously (SC) [7]. Inappropriately high or a lack of emergency increased dosing during GC replacement therapy is the major cause for the increased morbidity and mortality in AI [51].

### 3.2. Growth Hormone (GH) Deficiency Replacement Treatment

When GHD is suspected, GH stimulation tests are usually essential to confirm the deficiency as compared to single GH measurements. However, biochemical testing may not be required in patients with clear-cut features of GHD and three other documented pituitary hormone deficiencies: in patients with a clear pituitary lesion and a markedly subnormal insulin-like growth factor (IGF)1, a stimulation test may also not be absolutely necessary. GH replacement is then administered to patients with no contraindications at a starting dose of 0.2–0.4 mg/day for patients younger than 60 years and 0.1–0.2 mg/day for patients older than 60 years. GH doses are titrating to maintain IGF-1 levels above the mean/median for that age and sex group, but below the upper limit of normal (ULN), reducing the dose if AEs appear. Elderly adults with age-adjusted low IGF-1 levels and no history of pituitary or hypothalamic disease are not candidates for GH replacement. Starting doses should be increased slowly since fluid retention, a well-documented AE, depends from dose. Females display relative GH resistance, requiring higher starting and maintenance GH doses, similar to women receiving oral oestrogens. Persons with morbid obesity may also require increased GH dosing. The clinical effects of appropriate GH replacement are usually manifest within 6 weeks of initiating therapy, but may require a longer time period for maximum benefit [7]. In 509 adults with GHD (AGHD) (47% females) enrolled in the observational German NordiWin study over the years 2003–2013, it was confirmed that females and younger patients need higher GH doses when compared to males and older patients [52].

The more recent guidelines date back to 2011 and recommend GH dosing regimens to be individualised rather than previously-used weight-based regimens, starting with low doses and titrated according to clinical response, AEs, and IGF-I levels [9]. GH dosing should also take gender, oestrogen status, and age into consideration. After GH treatment initiation, monitoring is performed at 1- to 2-month intervals and then at 6-month intervals with a clinical evaluation and assessment for AEs, IGF-I levels, and clinico-metabolic parameters of GH response.

GH replacement AEs at the recommended doses (fluid retention, arthralgias, myalgias, paresthesias, carpal tunnel syndrome, sleep apnoea, sleep disturbances, dyspnoea) are reported in approximately 20% of patients but remit on lowering the GH dose. However, high doses of replacement may result to insulin resistance and/or new-onset diabetes [53]. Other suggested complication includes the development of new primary cancers but long-term studies did not confirm an increased prevalence or recurrence of the primary tumour after GH replacement therapy, and the occurrence of new tumours, if it occurs, appears to be very rare [53,54,55].

Another issue of concern is the compliance rate in GH replacement therapy, more a problem in the paediatric and adolescent population [56]; an increased compliance was seen when patients used a specific electronic device, the easypod™ Clinical Kit, with automated-injection and a docking station for recording objectively the administration data [57]. In this latter study, adherence to treatment was seen in 53 of 79 prepubertal patients with GHD [57]. In order to increase compliance, new compounds for GH replacement treatment are under investigation aiming to increase intra-injection intervals thus resulting in increased convenience and improved compliance, particularly in the context of the paediatric population [58]. Somapacitan, a reversible albumin-binding GH derivative, was compared in once-weekly regimen with once-daily Norditropin in a 26-week randomised, controlled, phase 3, safety and tolerability trial in AGHD patients [59]. The mean IGF-I standard deviation score (SDS) was similar for both formulations, whereas mild or moderate and transient AEs were seen for both drugs but somapacitan was associated with better treatment satisfaction. A C-terminal peptide-modified human GH (MOD-4023) given in a once-weekly scheme in GHD, adults and children, confirmed both efficacy and safety in a phase 2 study [60]. In a multicentre, phase 2, open-label, randomised, controlled study, safety, tolerability, efficacy, pharmacokinetics and pharmacodynamics of three different once-weekly doses of MOD-4023were compared to once-daily recombinant human GH (r-hGH) in prepubertal children with GHD for 12 months of treatment, revealing the better efficacy of 0.66 mg/kg/week dose along with a good tolerability and safety [61]. In a randomised, open-label, active-controlled study of 3 doses of once-weekly TransCon GH, a long-acting sustained-release r-hGH prodrug, compared to once-daily Genotropin; both drugs were comparable in the context of safety and efficacy [62]. Finally, in a phase 2 and 3, open-label, multicentre, randomised studies with a long-acting PEGylated r-hGH (0.2 mg/kg/week), when compared to once-daily rhGH (0.25 mg/kg/week) for 25 weeks in children with GHD, the former was shown to be effective and safe for GHD treatment [63] (Table 3).

Finally, initiation of GH therapy may alter requirements for GC and thyroxine replacement: this is discussed in more detail below.

### 3.3. Central Hypogonadism in Males

In males, SHG may be confirmed by measuring serum testosterone, FSH, LH, and prolactin (PRL) after an overnight fast and before 10:00 in the absence of any acute or subacute illness. The replacement then targets fat mass, bone, libido, sexual function, energy levels, sense of well-being, muscle mass and strength. Testosterone replacement is similar to male patients with primary hypogonadism (PHG) and is suggested for adult males with clear biochemical deficiency when they do not have any contraindications in order to prevent testosterone deficiency signs and symptoms [7]. Other situations to avoid testosterone replacement therapy include men seeking fertility, the presence of a hormone-related cancer (breast or prostate cancer), or suspicion of prostate cancer and/or lower urinary tract symptoms, high levels of prostate specific antigen (PSA) (> 4 ng/mL or > 3 ng/mL in men with increased risk), uncontrolled obstructive sleep apnoea or heart failure, or recent (within the last 6 months) myocardial infarction or stroke, elevated haematocrit or thrombophilia [10]. Τhe testosterone level-target is the mid-normal range with any of the approved formulation, considering patient preference, pharmacokinetics, pharmacodynamics, AEs, cost and availability. An important tool for a good testosterone replacement treatment is to evaluate symptoms, AEs, and compliance. Serum testosterone, haematocrit concentrations and prostate cancer risk evaluation are used to check patients with SHT during the first year after initiating testosterone therapy [10]. According the current guideline, a cost-benefit discussion has to be addressed to hypogonadal men 55- to 69-year-old with a life expectancy > 10 years, particularly in the context of prostate cancer risk with assessment before starting testosterone treatment and 3 to 12 months post-therapy. Similarly, younger hypogonadal patients 40- to 69-year-old but at increased risk of prostate cancer should follow a similar assessment. Patients more than 65 years can receive testosterone replacement therapy in an individualised manner after discussion of the risks and benefits [65].

The most common formulation used worldwide is given IM as depot injection of testosterone ester, testosterone enantate or cypionate with a starting dose 150–200 mg every 2 weeks or 75–100 mg/week, with testosterone enantate usually given 250 mg IM every 3 weeks. Its disadvantage is that large peaks and troughs of serum testosterone result in fluctuations in symptoms such as mood, libido and energy levels, but treatment is inexpensive, can be self-administrated with a flexibility of dosing, and is generally well tolerated. During follow-up monitoring, testosterone levels are measured midway between injections with mid-interval testosterone > 600 ng/dL (24.5 nmol/L) or < 350 ng/dL (14.1 nmol/L) the dose and the frequency need to be accordingly adjusted [10] with some further adjustment according the trough values just before the next injection. A depot long-acting testosterone injection given IM has become available in a formulation of testosterone undecanoate (Nebido), 1000 mg IM, given initially then at 6 weeks, and thereafter every 3 months [1,65], maintaining testosterone levels in normal range with minor fluctuations. Its disadvantage is that it has to be injected in a large volume of 4 mL IM, and coughing episodes have been reported after injection in a small proportion of patients [10]. During follow-up monitoring, the testosterone level-target at the end of the dosing interval just prior to the next injection is in the low-normal/subnormal range. The oral formulation of testosterone undecanoate is administered as 80 or 120 mg two or three times daily with fatty meals; it is metabolised to dihydrotestosterone in the intestine, being absorbed via the lymphatic system. It is well tolerated and it is most useful in patients with partial HGT or with intolerance to depot injections. However, it presents variability in its pharmacokinetic profile and consequently in its clinical outcomes, and it affects liver metabolism and lipid profiles. Testosterone levels are monitored at 3–5 h after ingestion with a fat-containing meal, but it is rare to obtain testosterone levels within the normal range, and there is uncertainty as to its effect on bone metabolism. Transdermal gel formulations have been commonly used since they provide flexibility of dosing, ease of application, good skin tolerability and possibly less erythrocytosis than injectable testosterone. Their disadvantages are referred to potential of transfer to other persons (females or children) by direct skin-to-skin contact, and skin irritation in a small proportion of treated men. Testosterone and dihydrotestosterone concentrations are moderately increased: the current formulations are of 1%, 1.62%, or 2% with doses 50–100 mg, 20.25–81 mg, and 40–70 mg applied to skin daily. In this case, the testosterone level is evaluated 2–8 h following the application, and with a treatment duration of at least 1 week. Buccal testosterone is formulated in bioadhesive tablets and they are placed on the buccal mucosa above the incisor tooth, allowing a slow release of testosterone over 12 h, but disadvantageous gum-related AEs have been reported. Testosterone levels are assessed immediately before or after application of a fresh system. Testosterone pellets containing 600–1200 mg testosterone are implanted SC, applied usually from 3 to 6 months (depending on the formulation). Despite its infrequent administration it requires surgical incision for insertion and a local haematoma or infection may occur, and rarely pellets may extrude spontaneously. During follow-up the testosterone level is measured at the end of the dosing interval with the dosing interval accordingly adjusted. Several other formulations such as testosterone axillary solution or nasal testosterone gel are being developed, targeting ease in application and discretion [1].

In patients wishing to become fertile, after discontinuation of testosterone replacement treatment, spermatogenesis can be initiated and maintained by gonadotropin therapy [human chorionic gonadotropin (hCG), human menopausal gonadotropin (hMG) or purified or recombinant FSH. When the abnormality emerges from the hypothalamic level, an additional option includes the stimulation by pulsatile gonadotropin-releasing hormone (GnRH). Both schemes have to be administered on average for 7–10 months, but fertility has been maintained to last up to 46 months in particular cases until pregnancy is achieved [66].

The marker for adequate replacement in the context of under- or overtreatment are serum testosterone levels that should be remain into mid-normal range adjusting according to the symptoms and biochemical markers. There is no evidence that the prostate carcinoma in patients on testosterone replacement is greater than background population risk [1]. Referral to an urologist is recommended if the patient has prostatic symptoms or elevated serum PSA increasing > 1.4 ng/mL over 12 months. It is also important to monitor the hematocrit and in case of polycythaemia detection, treatment has to be discontinued till the haematocrit becomes normal, whereas phlebotomy may be needed to prevent cardiovascular events [1]; careful dose titration may prevent this complication.

In one recent cohort of a large series of 40 patients with combined pituitary hormone deficiency, pulsatile GnRH therapy restored pituitary–testis axis function in approximately 60% of them, implying that pulsatile GnRH therapy may be equal or superior to combined gonadotropin therapy, at least in some patients, since more physiologic levels of gonadotropins are produced. However, intact gonadotroph cells are necessary for this action. Induction of spermatogenesis and virilisation has been described in treated patients [67]. Testosterone levels increase in some, but not in all, cases, indicating the need of a treatment lasting more than 3 months in specific cases [68,69]. A useful predictor index for the response to treatment was suggested from this later study, since all patients with LH levels > 3.0 IU/L after 1 month of treatment were responders [68].

### 3.4. Central Hypogonadism in Females

In females, SHG is confirmed by measurement of oestradiol (E2), FSH, and LH, assessment in the presence of oligomenorrhoea or amenorrhoea after excluding pregnancy and other causes of menstrual abnormality (such as hyperandrogenaemia, hyperprolactinaemia, thyroid disorders), particularly when no other pituitary hormone deficiency has been documented. One should also consider hypothalamic amenorrhoea due to weight loss, excessive exercise or stress. In post-menopausal women, the absence of an elevated serum FSH and LH (inappropriately-low) is sufficient for a diagnosis of gonadotroph dysfunction. In pre-menopausal women with SHG, hormonal replacement therapy (HRT) as oral oestrogen (unopposed oestrogens only for women who have undergone hysterectomy), or combined oestrogen and progestogen therapy (women with an intact uterus to prevent endometrial hyperplasia) is recommended, assuming that no contraindications (particularly related to breast carcinoma or thrombosis) are present [7]. The goals of treatment differ according to age: in older women the indication is the control of hot flushes and not to prevent the harm that the oestrogen deficiency carries as in the case of young women. Specifically, oestrogen is administrated daily with co-administration of progesterone for 12–14 consecutive days during a 4-week cycle; menses occur cyclically after progesterone withdrawal. A continuous regimen in which oestrogen and progesterone are combined may be associated by unpredictable menstrual bleeding the first months of therapy [1]. HRT is very effective in alleviating vasomotor symptoms of hypoestrogenism (hot flushes and night sweats), improving vaginal atrophy, urinary frequency and dysuria. Treatment with oestrogens until the age of 45 years or longer may reduce the risk of cardiovascular disease and mortality, along with bone mineral density impairment [1,7]. The combined oestrogen-progestin contraceptive pill seems to more acceptable by the younger females. The available oestrogen formulations have variable potency, AEs, cost, tolerability (oral, transdermal, topical gels and lotions, intravaginal creams and tablets, vaginal rings). The disadvantage of oral oestrogen is the extensive hepatic first-pass metabolism necessitating average doses of 1–2 mg E2, or equivalent, daily. Transdermal preparations are usually applied twice-weekly and provide 50-100 μg of E2 daily in a cyclical combination with a progestogen. Its disadvantage is possible skin irritation, as opposed to its advantage being the treatment of choice in patients with complex pituitary disease, since it avoids the effects of oral oestrogen on other hormone binding proteins and mimic better the physiological release of oestrogen to the systemic circulation as is seen with normal intact ovarian secretion [7,70]. Furthermore, in women on concomitant GH replacement, IGF-I generation is greater when transdermal rather than oral oestrogen is used, thereby decreasing the dose of GH required. Subcutaneous implants inserted on a 6-month basis may show tachyphylaxis and are difficult to reverse [11]. The choice of the oestrogen (and progestin) preparation relies on the risk of AEs, cost, and patient convenience [7].

Recently, it was documented in 184 consecutive women with GH replacement therapy that the oestrogen replacement treatment rate in women ≤ 52 years was lower compared to the replacement rates of the other pituitary hormone deficiencies (74% versus 100%); androgen replacement treatment was even lower in these women [71].

In patients with combined deficiencies such as gonadotrophin and ACTH deficiency, poor libido may be a problem due to androgen deficiency despite oestrogen and progesterone replacement [1]. Women with hypopituitarism with concomitant AI have a more severe androgen deficiency than those with PAI. Τhe recent guidelines recommended against the routine use of dehydro-epiandrosterone (DHEA) and testosterone in women with hypopituitarism [12]. However, a recent systematic review and meta-analysis demonstrated the short-term efficacy in sexual function improvement, and safety, of transdermal testosterone in naturally and surgically-menopausal women affected by hypoactive sexual desire disorder with or without co-administration of HRT [72]. DHEA is an androgen synthesised by the adrenal cortex and is regulated also by ACTH [1]. The treatment dose of DHEA used in most studies is between 25 and 50 mg once daily. The first randomised controlled trial of DHEA in 24 female patients with PAI or SAI showed that DHEA 50 mg daily for 4 months led to an improvement in sexual function, decreased depression scores and improved well-being when compared with placebo [73]. DHEA supplementation in hypopituitarism has shown benefit in terms of well-being and sexual function [74]. However, further studies with DHEA replacement in patients with AI, did not show similarly positive results in terms of sexual function and QoL [75]. In patients on GH replacement therapy, DHEA augments the IGF-I response thus resulting in decreased GH doses in females [76]. Clinical assessment of well-being, skin and axillary and pubic hair, as well as plasma DHEA sulfate (DHEAS) trough levels and plasma testosterone, can be measured to monitor DHEA dose [51,77]. Morning serum DHEAS levels should be measured before the intake of the daily dose, aiming at the mid-normal range [25]. Currently, DHEA is considered as a food supplement. The AEs of DHEA replacement include greasy skin, acne, hirsutism, alopecia, itching scalp/skin and increased sweat odour when the dose is too high, occurring not infrequently in elderly women but are generally responsive to dose reduction [51]. DHEA replacement could be tried in patients with persistent and seriously impaired QoL and reduced libido despite optimised conventional GC and mineralcorticoid replacement. However, when no positive subjective effects are experienced by the patients within 3 months, the therapy may be discontinued [51].

FSH and LH or their equivalent (recombinant forms) are administered by injections and are necessary for fertility. Reduced fertility outcomes in the context of ovulation per cycle or achievement of live-birth have been reported in some [78], but not in all studies [79].

### 3.5. Thyroid-Stimulating Hormone (TSH) Deficiency Replacement Treatment

The recent guidelines suggest measuring serum free T4 (fT4) and TSH to evaluate SHT. A fT4 level below the laboratory reference range in conjunction with a low, normal, or mildly elevated TSH (inappropriately low-TSH) in the setting of pituitary disease usually confirms a diagnosis of SHT.

In general, levothyroxine (L-T4) replacement in SHT is similar to that in primary hypothyroidism [70], but serum TSH cannot be used to assess adequacy of replacement. L-T4 has a long half-life, allowing once-daily dosing but difficulties in its absorption suggest administration fasting, whereas absorption is also inhibited by proton pump inhibitors. Novel forms, soft gel or liquid preparations allow its administration without food restriction. The goal of treatment is to keep fT4 levels in the mid-to-upper half of the reference range. This target is mostly achieved in average by L-T4 doses 1.6 μg/kg/d [80,81], with dose adjustments affected by age, weight, co-morbidities and physical activity, since TSH is not available as a marker of L-T4 replacement. Triiodothyronine (L-T3) displays better gastrointestinal absorption, and it can be used for short periods of time for dose initiation, but it is not generally used for long-term replacement [7,80]. Other formulations of thyroid hormones, such as thyroid extracts, are not recommended [7].

### 3.6. Prolactin (PRL) Deficiency

PRL deficiency is defined by its levels the below the normal reference range [70]. Since the major role of PRL is limited to lactation post-delivery, it was only investigated in one study subcutaneous injection of recombinant human PRL (60 μg/kg) in a twice-daily administration every 12 h in mothers with PRL deficiency. At the end of the 28-day study, PRL increased significantly, and milk volume increased from 3.4 ± 1.6 to 66.1 ± 8.3 mL/d with changes in milk composition similar to normal lactogenesis [82,83].

### 3.7. Central Diabetes Insipidus (CDI)

Central DI (CDI) is due to the partial or complete absence of posterior pituitary hormone AVP, also known as ADH, resulting in uncontrolled diuresis which can be life-threatening if not recognised. The diagnosis of DI is made by assessment of serum and urine osmolarity in the presence of polyuria (> 50 mL/kg body weight (BW)/24 h corresponding to 3.5 L/day in a 70-kg person). When serum osmolarity is elevated (> 295 mOsmol/L), urine osmolarity should be at least 600 mOsmol/L (urine osmolarity-to-plasma osmolarity ratio ≥ 2), after exclusion of glycosuria. Recently, the diagnostic performance of the test measuring copeptin, the C-terminal segment of the AVP prohormone which can be precisely measured as opposed to AVP *per se*, osmotically stimulated either by water deprivation or by hypertonic saline infusion, was proven to be superior to the classic indirect water-deprivation test in distinguishing CDI from primary polydipsia in patients with hypotonic polyuria [84]. Further studies are awaited. 1-desamino-8-D-arginine-vasopressin (desmopressin or DDAVP), is the synthetic analogue of AVP and its use results in effective antidiuresis after intranasal or oral administration in patients with CDI with individualized therapeutic schemes. A therapeutic trial of DDAVP, 10–20 μg intranasally may control polyuria for up to 16 h. Complete AVP deficiency can lead to polyuria exceeding 10 L/24 h. When the thirst mechanism is working well and there is free access to water, patients can maintain water balance by increased drinking. A suggested and safe approach is represented by mild undertreatment, since water homeostasis can still be maintained by thirst mechanisms. Patients with partial DI who can tolerate polyuria may choose no treatment. It has been stated that 25% of DI patients with an intact thirst mechanism treated with *DDAVP* had mild hyponatraemia due to the inability to reverse the antidiuretic effect of the drug when fluid intake exceeds requirements [7,85]. A once-weekly phase of polyuria may further decrease this risk. If DI is accompanied by a reduced thirst threshold, resulting in adipsic hypernatraemia, it is most important to monitor BW and urine output on a fixed dose of DDAVP and adjust fluid intake as appropriate. In the outpatient setting in patients with an intact thirst mechanism, clinicians should administer the lowest dose of DDAVP dose that results in minimal disruption of sleep at night with and minimal disruption of activities at daytime. In cases of mild DI, many patients will demonstrate adequate control with a single bedtime dose of intranasal DDAVP but an additional morning dose may be required.

For follow-up monitoring, sodium, urea, creatinine, uric acid, hypokalemia and the presence or the absence of polyuria, all need to be assessed. In cases of adipsic DI, DDAVP and fluid intake have to be carefully titrated, with frequent weighing and serum sodium level monitoring. Following pituitary surgery, attempts to discontinue DDAVP should be attempted to determine whether posterior pituitary function has recovered. Some suggest that all patients with DI should wear a bracelet to protect them in case of emergency [7].

DI represents a life-threatening entity when the thirst mechanism is impaired, as in cases of hypothalamic disorder or reduced consciousness or lack of water availability such as in cases of physical disability resulting in severe dehydration and death. For this reason, a Society for Endocrinology guideline has been recently issued for the in-patient management of patients with CDI [7,86].

Treatment AEs includes hyponatraemia due to overdosing. This complication is more problematic in the elderly who may have increased renal sensitivity to the drug and/or may have abnormalities in osmoregulation. The thirst mechanism may be altered by damaged hypothalamic osmoreceptors, resulting in a high risk of both hypernatraemia and hyponatraemia since patients cannot adjust fluid intake according to thirst. As noted above, a fixed dose of DDAVP and a constant amount of fluid intake together with consistent environmental temperature and humidity conditions are suggested to result in ‘water-balance’.

### 3.8. Considerations Regarding Combinations of Hormone Replacement Therapies

In cases of simultaneous AI and thyroid dysfunction, GC replacement should be started first to avoid precipitating acute AI [13]. When there is no time to wait for a diagnosis, empiric GC therapy may be given along with starting L-T4 therapy taking blood for a later measurement of cortisol until there is a definitive laboratory confirmation of cortisol reserve. Thyroid hormone accelerates endogenous cortisol clearance, and patients with low adrenal reserve may be rendered hypoadrenal precipitating an AC. Moreover, physiological and pharmacological doses of GC suppress TSH levels. In addition, cortisol sufficiency has to be tested before and after GH replacement initiation. Patients with GHD demonstrate an increased cortisol/cortisone metabolite ratio, whereas since GH suppresses the conversion of cortisone to cortisol patients starting GH replacement therapy may need increased doses of GC replacement therapy [7]. In patients on GH replacement therapy, DHEA augments the IGF-I response thus resulting in decreased GH doses in females [76]. When not already treated, AI may become apparent because of reduced adrenal reserves. Moreover, in women taking HRT, oestrogen-increased CBG results in increased total serum cortisol levels, but not the unbound active fraction. This can be avoided by using transdermal oestrogen therapy. Similarly, an adjustment in L-T4 therapy and an increase in GC doses may be needed in women starting HRT or an increase in GC doses may be needed in both females and males starting HRT. Oestrogen-dependent liver production of thyroid-binding globulin (TBG) result in increased requirements for L-T4 dose requirements from 1.3 to 1.8 μg/kg/d [7].

In addition, thyroid replacement treatment should be checked and possibly increased in patients before GH replacement therapy, since patients with a borderline SHT may necessitate immediate treatment or within 3–6 months of GH replacement therapy initiation. In one study, only men with SHT required increased L-T4 doses after starting GH therapy [87]. Conversely, abnormal GH and IGF-1 secretion occur in hypothyroidism. Finally, AI may mask the presence of partial DI and monitoring for the development of DI is needed after the initiation of GC replacement; similarly, when DI improves without any intervention AI has to be excluded: since deficits in GC causes reduced free renal water clearance, a borderline polyuria caused by DI may be masked [7] (Table 4).

## 4. Considerations Concerning Specific Populations with Hypopituitarism

### 4.1. Pregnancy

Woman with AI usually do not need to alter GC doses during the first two trimesters of pregnancy. An increase by 20–40% in the dosing of HC maybe needed during the third trimester since free cortisol normally increases during this period [7,51,88]. Lower doses of HC might be needed in women with hypopituitarism who have a borderline cortisol reserve.

It is very important to closely follow-up pregnant women with AI in order to document clinical symptoms and signs of GC over- and under-replacement by measuring weight gain, postural hypotension or hypertension, hyperglycaemia and fatigue. Only HC should be used in pregnancy since it is degraded by the enzyme 11β-HSD1, and does not cross the placenta, as opposed to dexamethasone which has to be avoided since it is not inactivated in the placenta. During the active phase of labour, doses should be adjusted to that used in major surgical stress by administering 50 mg HC intravenously (IV) in the second stage of labour. For Caesarean section, a dose of 50–100 mg every 6–8 h is recommended.

Thyroid replacement should be monitored every 4–6 weeks since increased L-T4 doses may be required to maintain serum fT4 or total T4 levels within target ranges [7]. Current recommendations include increasing L-T4 doses by doubling the dose at weekends on confirmation of pregnancy with further dose adjustments to achieve target levels. Since many fT4 assays do not perform well during pregnancy, it is suggested to use total T4 reference ranges adjusted upward by 50% if pregnancy-specific fT4 reference ranges are not available. Clinicians should reduce L-T4 doses back to pre-pregnancy levels immediately after delivery to avoid iatrogenic hyperthyroidism [7].

In pregnant women with DI, it is suggested to continue DDAVP administration during pregnancy, adjusting the dose if required, since the different water-balance which is established by a decreased threshold for AVP release and thirst mechanism results in decreased serum sodium and osmolality [89]. Moreover, the enzyme vasopressinase, which degrades endogenous AVP, is increased as it is produced by the placenta. However, pregnancy may unmask mild forms of DI, often recurring with subsequent pregnancies. Usually, the requirement for DDAVP does not change but it sometimes might be slightly higher [90]. DDAVP is generally considered safe during pregnancy and for the newborn in lactating mothers [7,89]. GH replacement is discontinued during pregnancy because there is no evidence as yet for its safety, whereas the placenta produces GH [7].

### 4.2. Pituitary Apoplexy

Classical pituitary apoplexy (PitAp) may be a life-threatening situation and rapid replacement with HC may be life-saving. PitAp can be caused either by haemorrhage and/or by infarction of a tumour within the pituitary gland followed by a clinical syndrome which includes the sudden onset of a headache along with vomiting, visual disturbances and neurological symptoms including reduced consciousness or impairment of cranial nerves. A high index of clinical suspicion is essential since prompt management is not only life- but additionally sight-saving [6]. When PitAp is confirmed, it ideally should be referred to a multidisciplinary team (MDT) comprising both neurosurgical and endocrinological expertise since decision management may often be difficult [81], and in many cases it is not clear whether the best approach should be the conservative or surgical one. A wait-and-see approach may be appropriate, but in patients with severe and persistent deteriorating visual disturbances in the context of neuro-ophthalmic signs, visual acuity and field or with altered level of consciousness, may be switched to a surgical approach. Similarly, in the presence of a new visual or worsening neurological signs or symptoms, surgery should be urgently considered [91].

Follow-up management also has to include tumour monitoring to early identify tumour recurrence, hormonal evaluation to early identify pituitary insufficiency, and neurological examination to document any remaining neurological deficit [91].

In the acute setting, the care of patients with PitAp should include careful assessment of fluid and electrolyte balance, replacement of GCs and supportive measures for haemodynamic equilibrium [91]. Acute SAI is seen in approximately two-thirds of patients with PitAp and is the major source of mortality associated with the condition. Patients with PitAp may have nausea and vomiting, so oral GCs are not recommended in the acute setting. Hydrocortisone 100–200 mg as bolus administration given IV after taking blood for a later measurement of hormones, including cortisol, fT4, TSH, PRL, IGF1, GH, LH, FSH, testosterone and E2 (in men and women, respectively) and electrolytes, renal function, liver function, full blood count and a clotting screen. The next step includes a continuous infusion IV every 2–4 mg/h or 50–100 mg injection IM every 6 h. Intermittent IV administration is not recommended since much of the administered GC will be filtered into the urine. After recovery from the acute emergency, the HC dose should be quickly tapered to the maintenance dose. Cortisol sufficiency should be re-evaluated 2–3 months post-PitAp episode. As part of conservative, non-surgical management, dexamethasone can be used to reduce oedema [91]. Patients who do not fulfill the criteria for urgent empirical GCs therapy should be considered for treatment with oral GCs if their 09.00 h serum cortisol is < 450 nmol/L [91], but the precise threshold will depend on the assay in use and the degree of illness. Ideally, any surgical approach should take place within the first week of onset of symptoms, or in case with resistance to GC therapy after 1 week of treatment [91,92,93,94]. Retrospective studies did not find differences in the endocrine outcome between the surgical and conservative approaches [22,93,95]. All patients with PitAp should have ophthalmological and hormonal evaluation in the immediate post-operative or post-acute period and at 4–8 weeks and following the episode, and annually thereafter [91]. Approximately 80% of the patients will need hormone replacement therapy after PitAp [22,92,96]. GHD represents the most common endocrine deficit, while GCs are replaced in 60–80%, thyroid hormone in 50–60%, DDAVP in 10–25% of patients and testosterone in 60–80% of men [24,93,95,96]. Significantly, in a series of 45 patients, 20% in the surgically-treated and 11% in the conservatively managed had normal pituitary function at follow-up [92].

### 4.3. Adrenal Crisis

When AI is presents acutely, it may be a life-threatening medical emergency. AC is presented particularly in the context of conditions with increased needs such as serious infection, acute stress, surgery, trauma, bilateral adrenal infarction, or haemorrhage. Retrospective and prospective analysis revealed a prevalence of 6.6–8.3 AC/100 patient-years, with mortality 0.5/100 patient-years, mainly due to gastrointestinal and other infectious diseases [25].

The recently issued guidance from the Society for Endocrinology recommends on suspicion of hypoadrenalism drawing a serum sample for cortisol, immediately followed by the administration of bolus injection of 100 mg HC IV or IM followed by continuous infusion IV of 200mg HC/24 h (or 50 mg HC injection IV or IM every 6 h), followed by rehydration with the rapid infusion IV of 1000 mL of isotonic saline infusion within the first hour, and finally, followed by further rehydration IV as required, usually 4–6 L in 24 h along with monitoring for fluid overload in case of renal impairment and in elderly patients [97]. Hypoglycaemia when present should be also treated by a slow dextrose infusion IV [25].

The prevention of AC is of major importance. The ‘sick-day rules’ represents the most important part of patient education. During minor illness or minor surgery, it is suggested a dose increase up to 2–3 times the normal daily dose for 2–3 days. During major illness or major surgery, it is suggested to be covered with HC 50–100 mg 6-hourly parenterally, then tapering down to a double normal oral dose daily for a couple of days, contingent upon full consciousness and eating and drinking normally. Regarding uncomplicated, outpatient dental procedures under local anaesthesia and most radiologic studies, no additional dosage is suggested. In the event of a severe stress or trauma, use of a prefilled syringe for injection is recommended. In the event of moderately stressful procedures (barium enema, endoscopy, or arteriography) an IV administration of 100 mg HC is recommended just before the procedure [25].

### 4.4. Pituitary Metastases

The increase in the lifespan of cancer patients has resulted in an increased prevalence of metastases to otherwise rare sites. Pituitary metastases have been described in a range 3–27% in autopsy series in the context of disseminated malignancy [98], occurring mostly in patients with breast (37.2%) and lung (24.2%) cancer [18,99,100]. Panhypopituitarism (27.7%) and DI (27.7–70%) were between the most common clinical presentation following visual abnormalities [18,100]. When DI presents rapidly in patients over 50 years of age, pituitary metastases has to be considered in the differential diagnosis even in the absence of an obvious primary [101], noting that symptoms may be masked by ACTH deficiency and become more evident after GC replacement therapy [102]. PitAp may also occur with a pituitary metastasis or a metastasis may be located in a pituitary adenoma [96]. Regarding the management of hypopituitarism due to a pituitary metastasis, deficient hormone replacement treatment should be given together with surgery, radiosurgery, radiation therapy and chemotherapy, as appropriate [19,101].

## 5. Future Perspectives of Hypopituitarism Managements

As mentioned previously, the task of replacement therapies in hypopituitarism is to minimise the symptoms and the signs of each hormone deficiency with safety, namely, minimising AEs and maximising QoL. In order to achieve and mimic the physiological secretion of the deficient hormones it is important to achieve the appropriate dose but also the appropriate timing of hormones replacement. The importance of the timing and profile of hormonal secretion has been recently documented in patients with AI treated with the once-daily MRHC who showed a similar profile in clock gene expression compared with the patients treated by the standard multiple daily-doses GC replacement therapy that exhibited dysregulation in clock gene expression [103] Future studies optimising replacement with maximal patient convenience are to be anticipated.

## 6. Conclusions

The management of pituitary insufficiency focuses on two important issues. The first refers to the immediate clinical suspicion particularly in situations of ACTH and TSH- insufficiency, since their lack that may be life-threatening. The second important issue refers to the magnitude of replacement, to the ease of replacement, and finally to the ability to mimic the physiological rhythm of secretion, where necessary. Nowadays, research focuses on the improvement of GC delivery to better match the physiological circadian clock of cortisol secretion, and on the development of long-lasting formulations of GH in order to substitute the daily injections with weekly injections. The establishments of MDTs to treat these patients along with the guidelines that have been issued in the context of hormonal deficiencies should enable the uniform management of hypopituitarism, making possible the comparison of variable therapies and their outcomes in order to improve the available treatment options.

## Figures and Tables

**Table 1:** Etiology of hypopituitarism.

**Congenital [1,7,16]**
**Isolated pituitary hormone deficiency [1,7]**
KAL, DAX-1, GH-1, GnRH, GHRH and TRH receptor mutations Prader-Willi and Bardet-Biedl syndromes
**Single or multiple pituitary hormone deficiency [1,7]**
PIT-1, PROP-1, HESX-1, SOX 2 mutations
**Neoplastic [1,7,17,18,19,20]**
Pituitary adenoma (Functioning and non-functioning) Craniopharyngioma Meningioma Cysts (Rathke’s cleft, arachnoid, epidermoid, dermoid) Germinoma Glioma Astrocytoma Ganglioneuroma Paraganglioma Teratoma Chordoma/Chondrosarcoma Pituicytoma Ependymoma Pituitary carcinoma Metastases
**Infiltrative/Inflammatory/Immunological [1,7,13,21]**
Autoimmune (lymphocytic hypophysitis, pituitary and POUF-1 antibodies) Granulomatous (granulomatosis with polyangiitis, sarcoidosis) Xanthomatous hypophysitis Necrotising hypophysitis IgG4-related hypophysitis Sarcoidosis Haemochromatosis Langerhans cell histiocytosis Giant cell granuloma
**Infectious [1,7]**
Bacterial Fungal Parasites Tuberculosis Syphilis
**Vascular [1,7,22]**
Pituitary apoplexy Sheehan’s syndrome Intrasellar carotid artery Aneurysm Subarachnoid haemorrhage
**Traumatic [1,7,23]**
Head injury
**Empty sella [1,7,24]**
**Medications [1,7,25,26]**
Opiates (primarily gonadotropins, ACTH, GH) GCs (ACTH only) Megestrol acetate (ACTH only) Somatostatin analogues (GH, ACTH, TSH) CTLA-4 blockers (ACTH, TSH, LH/FSH)
**Iatrogenic [1,7,14,26,27]**
Surgery Radiotherapy (Pituitary, nasopharyngeal, cranial) Immune Checkpoint Inhibitors (CTLA-4 blockers)
**Idiopathic [1,7]**

**Table 2:** Compounds that have been used for the adrenal insufficiency.

Glucocorticoid	Time of Administration	Usual Doses
Hydrocortisone	2–3 times daily; early morning, early afternoon, 6 h away from bedtime	15–20 mg
Prednisolone	Once daily; morning	3–5mg
Modified-release hydrocortisone (Plenadren)	Once daily; early morning	Equal to hydrocortisone or a dose increased at 20% compared with hydrocortisone
Modified-release hydrocortisone (Chronocort)	2 times daily; before bedtime and early morning	20 mg at 23:00 and 10 mg at 07:00
Oral immediate-release granule formulation of HC (Alkindi)	Once daily; early morning	Equal to hydrocortisone but appropriate dosing for the children

**Table 3:** New compounds in long-acting forms in of growth hormone replacement treatment [64].

Novel GH Long-Releasing Drug	Characteristics of the Formulations
Somapacitan	Reversible albumin-binding GH derivative
MOD-4023; GX-H9; LAPSrhGH/HM10560A; ProFuse GH	GH fusion protein
TransCon ACP-001	long-acting sustained-release r-hGH prodrug
Jintrolong; BBT-031	Long-acting PEGylated r-hGH
LB03002; CP016	Depot
AG-B1512	Fab antibody-binding GH molecule

GH: Growth Homone; PEG-rhGH: Pegylated recombinant human growth hormone; r-hGH: recombinant human growth hormone.

**Table 4:** Treatment in combined hormonal deficiencies and doses adjustments [7].

Hormone Deficiencies	1st Drug Introduced	2nd Drug Introduced	Rationale
Cortisol and TSH	GC	L-T4	Thyroid hormone accelerates endogenous cortisol clearance
Cortisol and GH	GC	GH	Increased cortisol/cortisone metabolite ratio in GHD and GH suppresses cortisone to cortisol conversion
AVP and cortisol	GC	DDAVP	GC deficiency induces impaired free renal water clearance covering the polyuria of ADH deficiency
	Doses adjustments		
Gonadotrophin and TSH or cortisol	Increase in L-T4 or GC after treatment initiation of HRT		Estrogen-dependent liver production of thyroid-binding globulin (TBG) and corticosteroid-binding globulin (CBG)
GH and TSH	Increase in L-T4 after treatment initiation of GH replacement therapy		36–47% Euthyroid patients and 16–18% of hypothyroid patients developed low fT4 levels within 3–6 months of GH therapy initiation; IGF-1 levels are reduced in hypothyroid patients and GH stimulation tests may be blunted
GH and oestrogen	Increase in GH doses after treatment initiation of HRT		Oestrogens ↓ circulating IGF-1 levels in GHD (↑ postprandial lipid oxidation and ↓ protein synthesis antagonizing the metabolic actions of GH).
GH and DHEA	DHEA augments the IGF-I response		↓ GH doses (females)

ADH: antidiuretic hormone; DDAVP: desmopressin; IGF: insulin-like growth factor; GC: glucocorticoid; GH: growth hormone; GHD: growth hormone deficiency; HRT: hormone replacement treatment; L-T4: levothyroxine; TSH: thyroid-stimulating hormone.

## Abbreviations

AC	adrenal crisis;
ACTH	adrenocorticotropic hormone;
ADH	antidiuretic hormone;
AE	adverse event;
AGHD	adults with growth hormone deficiency;
AI	adrenal insufficiency;
AVP	arginine vasopressin;
BSA	body surface area;
BW	body weight;
CBG	corticosteroid-binding globulin;
CDI	central diabetes insipidus;
CSHI	continuous subcutaneous hydrocortisone infusion;
CTLA-4	cytotoxic T-lymphocyte antigen-4;
DDAVP	1-desamino-8-D-arginine-vasopressin or desmopressin;
DHEA	dehydroepiandrosterone;
DHEAS	dehydroepiandrosterone sulfate;
DI	diabetes insipidus;
DM	diabetes mellitus;
E2	oestradiol;
ICI	immune checkpoint inhibitors;
IGF	insulin-like growth factor;
IHD	isolated deficiency;
IM	intramuscularly;
IV	intravenously;
FSH	follicle stimulating hormone;
fT4	free T4;
GC	glucocorticoid;
GH	growth hormone;
GHD	growth hormone deficiency;
GnRH	gonadotropin-releasing hormone;
HC	hydrocortisone;
HCDC	hydrocortisone day-curve;
HCeq	equivalent hydrocortisone dose;
hCG	human chorionic gonadotropin;
hMG	human menopausal gonadotropin;
HRT	hormonal replacement therapy;
LH	Luteinizing Hormone;
L-T4	levothyroxine;
MDT	multidisciplinary team;
MPHD	multiple pituitary hormone deficiency;
MRHC	once-daily modified-release hydrocortisone;
NFPA	non-functioning pituitary adenoma;
PAI	primary adrenal insufficiency;
PD-1	programmed cell-death-1;
PD-L1	programmed cell-death-ligand 1 (PD-L1);
PHG	primary hypogonadism;
PitAp	pituitary apoplexy;
PRL	prolactin;
PSA	prostate specific antigen;
QoL	quality of life;
r-hGH	recombinant human growth hormone;
SAI	central or secondary adrenal insufficiency;
SC	subcutaneously;
SDS	standard deviation score;
SHG	secondary hypogonadism;
SHT	secondary hypothyroidism;
TBG	thyroid-binding globulin;
TSH	thyroid-stimulating hormone;
UFC	urinary free cortisol;
ULN	upper limit of normal;
11β-HSD1	enzyme 11β-hydroxysteroid dehydrogenase

## References

[1] Chung T.T., Koch C.A., Monson J.P., Feingold K.R., Anawalt B., Boyce A., Chrousos G., Dungan K., Grossman A., Hershman J.M., Kaltsas G., Koch C., Kopp P. (2018). Hypopituitarism. Endotext.

[2] Yuen K.C.J., Mattsson A.F., Burman P., Erfurth E.M., Camacho-Hubner C., Fox J.L., Verhelst J., Geffner M.E., Abs R. (2018). Relative Risks of Contributing Factors to Morbidity and Mortality in Adults with Craniopharyngioma on Growth Hormone Replacement. J. Clin. Endocrinol. Metab..

[3] Bülow B., Attewell R., Hagmar L., Malmström P., Nordström C.H., Erfurth E.M. (1998). Postoperative prognosis in craniopharyngioma with respect to cardiovascular mortality, survival, and tumor recurrence. J. Clin. Endocrinol. Metab..

[4] Olsson D.S., Bryngelsson I.L., Ragnarsson O. (2016). Higher incidence of morbidity in women than men with non-functioning pituitary adenoma: A Swedish nationwide study. Eur. J. Endocrinol..

[5] Burman P., Mattsson A.F., Johannsson G., Hoybye C., Holmer H., Dahlqvist P., Berinder K., Engström B.E., Ekman B., Erfurth E.M. (2013). Deaths among adult patients with hypopituitarism: Hypocortisolism during acute stress, and de novo malignant brain tumors contribute to an increased mortality. J. Clin. Endocrinol. Metab..

[6] Baldeweg S.E., Vanderpump M., Drake W., Reddy N., Markey A., Plant G.T., Powell M., Sinha S., Wass J., Society for Endocrinology Clinical Committee (2016). Society for Endocrinology Endocrine Emergency Guidance: Emergency management of pituitary apoplexy in adult patients. Endocr. Connect..

[7] Fleseriu M., Hashim I.A., Karavitaki N., Melmed S., Murad M.H., Salvatori R., Samuels M.H. (2016). Hormonal Replacement in Hypopituitarism in Adults: An Endocrine Society Clinical Practice Guideline. J. Clin. Endocrinol. Metab..

[8] Bornstein S.R., Allolio B., Arlt W., Barthel A., Don-Wauchope A., Hammer G.D., Husebye E.S., Merke D.P., Murad M.H., Stratakis C.A. (2016). Diagnosis and Treatment of Primary Adrenal Insufficiency: An Endocrine Society Clinical Practice Guideline. J. Clin. Endocrinol. Metab..

[9] Molitch M.E., Clemmons D.R., Malozowski S., Merriam G.R., Vance M.L., Endocrine Society (2011). Evaluation and treatment of adult growth hormone deficiency: An Endocrine Society clinical practice guideline. J. Clin. Endocrinol. Metab..

[10] Bhasin S., Brito J.P., Cunningham G.R., Hayes F.J., Hodis H.N., Matsumoto A.M., Snyder P.J., Swerdloff R.S., Wu F.C., Yialamas M.A. (2018). Testosterone Therapy in Men with Hypogonadism: An Endocrine Society Clinical Practice Guideline. J. Clin. Endocrinol. Metab..

[11] Stuenkel C.A., Davis S.R., Gompel A., Lumsden M.A., Murad M.H., Pinkerton J.V., Santen R.J. (2015). Treatment of Symptoms of the Menopause: An Endocrine Society Clinical Practice Guideline. J. Clin. Endocrinol. Metab..

[12] Wierman M.E., Arlt W., Basson R., Davis S.R., Miller K.K., Murad M.H., Rosner W., Santoro N. (2014). Androgen therapy in women: Areappraisal: An Endocrine Society clinical practice guideline. J. Clin. Endocrinol. Metab..

[13] Prete A., Salvatori R., Feingold K.R., Anawalt B., Boyce A., Chrousos G., Dungan K., Grossman A., Hershman J.M., Kaltsas G., Koch C., Kopp P. (2018). Hypophysitis. Endotext.

[14] Pekic S., Miljic D., Popovic V., Feingold K.R., Anawalt B., Boyce A. (2018). Hypopituitarism Following Cranial Radiotherapy. Endotext.

[15] Martins F., Sofiya L., Sykiotis G.P., Lamine F., Maillard M., Fraga M., Shabafrouz K., Ribi C., Cairoli A., Guex-Crosier Y. (2019). Adverse effects of immune-checkpoint inhibitors: Epidemiology, management and surveillance. Nat. Rev. Clin. Oncol..

[16] Xatzipsalti M., Voutetakis A., Stamoyannou L., Chrousos G.P., Kanaka-Gantenbein C. (2019). Congenital Hypopituitarism: Various Genes, Various Phenotypes. Horm. Metab. Res..

[17] Drummond J.B., Ribeiro-Oliveira A., Soares B.S., Feingold K.R., Anawalt B., Boyce A., Chrousos G., Dungan K., Grossman A., Hershman J.M., Kaltsas G., Koch C., Kopp P. (2018). Non-Functioning Pituitary Adenomas. Endotext.

[18] Javanbakht A., Badie B. (2018). Pituitary metastasis: A rare condition. Endocr. Connect..

[19] Angelousi A., Alexandraki K.I., Kyriakopoulos G., Tsoli M., Thomas D., Kaltsas G.A., Grossman A.B. (2019). Neoplastic metastases to the endocrine glands. Endocr. Relat. Cancer.

[20] Kaltsas G.A., Nomikos P., Kontogeorgos G., Buchfelder M., Grossman A.B. (2005). Clinical review: Diagnosis and management of pituitary carcinomas. J. Clin. Endocrinol. Metab..

[21] Makras P., Alexandraki K.I., Chrousos G.P., Grossman A.B., Kaltsas G.A. (2007). Endocrine manifestations in Langerhans cell histiocytosis. Trends Endocrinol. Metab..

[22] Randeva H.S., Schoebel J., Byrne J., Esiri M., Adams C.B., Wass J.A. (1999). Classical pituitary apoplexy: Clinical features, management and outcome. Clin. Endocrinol. (Oxf).

[23] Ntali G., Tsagarakis S. (2019). Traumatic brain injury induced neuroendocrine changes: Acutehormonal changes of anterior pituitary function. Pituitary.

[24] Miljic D., Pekic S., Popovic V., Feingold K.R., Anawalt B., Boyce A., Chrousos G., Dungan K., Grossman A., Hershman J.M., Kaltsas G., Koch C., Kopp P. (2018). Empty Sella. Endotext.

[25] Alexandraki K.I., Grossman A., Feingold K.R., Anawalt B., Boyce A., Chrousos G., Dungan K., Grossman A., Hershman J.M., Kaltsas G., Koch C., Kopp P. (2018). Adrenal Insufficiency. Endotext.

[26] Castinetti F., Albarel F., Archambeaud F., Bertherat J., Bouillet B., Buffier P., Briet C., Cariou B., Caron P., Chabre O. (2018). French Endocrine Society Guidance on endocrine side-effects of immunotherapy. Endocr. Relat. Cancer.

[27] Appelman-Dijkstra N.M., Kokshoorn N.E., Dekkers O.M., Neelis K.J., Biermasz N.R., Romijn J.A., Smit J.W., Pereira A.M. (2011). Pituitary dysfunction in adult patients after cranial radiotherapy: Systematic review and meta-analysis. J. Clin. Endocrinol. Metab..

[28] Oster H., Challet E., Ott V., Arvat E., de Kloet E.R., Dijk D.J., Lightman S., Vgontzas A., van Cauter E. (2017). The Functional and Clinical Significance of the 24-Hour Rhythm of Circulating Glucocorticoids. Endocr. Rev..

[29] Arlt W., Allolio B. (2003). Adrenal insufficiency. Lancet.

[30] Esteban N.V., Loughlin T., Yergey A.L., Zawadzki J.K., Booth J.D., Winterer J.C., Loriaux D.L. (1991). Daily cortisol production rate in man determined by stable isotope dilution/mass spectrometry. J. Clin. Endocrinol. Metab..

[31] Hahner S., Loeffler M., Fassnacht M., Weismann D., Koschker A.C., Quinkler M., Decker O., Arlt W., Allolio B. (2007). Impaired subjective health status in 256 patients with adrenal insufficiency on standard therapy based on cross-sectional analysis. J. Clin. Endocrinol. Metab..

[32] Bleicken B., Hahner S., Loeffler M., Ventz M., Decker O., Allolio B., Quinkler M. (2010). Influence of hydrocortisone dosage scheme on health-related quality of life in patients with adrenal insufficiency. Clin. Endocrinol. (Oxf).

[33] Swords F.M., Drake W.M., Carroll P.V., Monson J.P. (2004). Hormonal therapy for hypopituitarism. Encycl.Endocr. Dis..

[34] Alexandraki K.I., Grossman A.B. (2011). Is urinary free cortisol of value in the diagnosis of Cushing’s syndrome?. Curr. Opin. Endocrinol. Diabetes. Obes..

[35] Trainer P.J., Besser G.M. (1995). The Bart’s Endocrine Protocols.

[36] Ceccato F., Barbot M., Lizzul L., Selmin E., Saller A., Albiger N., Betterle C., Boscaro M., Scaroni C. (2018). Decrease in salivary cortisol levels after glucocorticoid dose reduction in patients with adrenal insufficiency: A prospective proof-of-concept study. Clin. Endocrinol. (Oxf).

[37] Raff H. (2016). Measurement of salivary cortisone to assess the adequacy of hydrocortisone replacement. J. Clin. Endocrinol. Metab..

[38] Isidori A.M., Arnaldi G., Boscaro M., Falorni A., Giordano C., Giordano R., Pivonello R., Pozza C., Sbardella E., Simeoli C. (2019). Towards the tailoring of glucocorticoid replacement in adrenal insufficiency: The Italian Society of Endocrinology Expert Opinion. J. Endocrinol. Invest..

[39] Oelkers W., Diederich S., Bahr V. (2001). Therapeutic strategies in adrenal insufficiency. Ann. Endocrinol. (Paris).

[40] Falorni A., Minarelli V., Morelli S. (2013). Therapy of adrenal insufficiency: An update. Endocrine.

[41] Smith D.J.F., Prabhudev H., Choudhury S., Meeran K. (2017). Prednisolone has the same cardiovascular risk profile as hydrocortisone in glucocorticoid replacement. Endocr. Connect..

[42] Johannsson G., Bergthorsdottir R., Nilsson A.G., Lennernas H., Hedner T., Skrtic S. (2009). Improving glucocorticoid replacement therapy using a novel modified-release hydrocortisone tablet: Apharmacokinetic study. Eur. J. Endocrinol..

[43] Isidori A.M., Venneri M.A., Graziadio C., Simeoli C., Fiore D., Hasenmajer V., Sbardella E., Gianfrilli D., Pozza C., Pasqualetti P. (2018). Effect of once-daily, modified-release hydrocortisone versus standard glucocorticoid therapy on metabolism and innate immunity in patients with adrenal insufficiency (DREAM): A singleblind, randomised controlled trial. Lancet Diabetes Endocrinol..

[44] Newell-Price J., Whiteman M., Rostami-Hodjegan A., Darzy K., Shalet S., Tucker G.T., Ross R.J. (2008). Modified-release hydrocortisone for circadian therapy: Aproof-of-principle study in dexamethasone-suppressed normal volunteers. Clin. Endocrinol. (Oxf).

[45] Debono M., Ghobadi C., Rostami-Hodjegan A., Huatan H., Campbell M.J., Newell-Price J., Darzy K., Merke D.P., Arlt W., Ross R.J. (2009). Modified-release hydrocortisone to provide circadian cortisol profiles. J. Clin. Endocrinol. Metab..

[46] Verma S., Vanryzin C., Sinaii N., Kim M.S., Nieman L.K., Ravindran S., Calis K.A., Arlt W., Ross R.J., Merke D.P. (2010). A pharmacokinetic and pharmacodynamic study of delayed- and extended-release hydrocortisone (Chronocort) vs. conventional hydrocortisone (Cortef) in the treatment of congenital adrenal hyperplasia. Clin. Endocrinol. (Oxf).

[47] Whitaker M.J., Debono M., Huatan H., Merke D.P., Arlt W., Ross R.J. (2014). An oral multiparticulate, modified-release, hydrocortisone replacement therapy that provides physiological cortisol exposure. Clin. Endocrinol. (Oxf).

[48] Porter J., With M., Ross R.J. (2018). Immediate-release granule formulation of hydrocortisone, Alkindi(R), for treatment of paediatric adrenal insufficiency (Infacort development programme). Expert Rev. Endocrinol. Metab..

[49] Oksnes M., Björnsdottir S., Isaksson M., Methlie P., Carlsen S., Nilsen R.M., Broman J.E., Triebner K., Kämpe O., Hulting A.L. (2014). Continuous subcutaneous hydrocortisone infusion versus oral hydrocortisone replacement for treatment of Addison’s disease: A randomized clinical trial. J. Clin. Endocrinol. Metab..

[50] Ragnarsson O., Mattsson A.F., Monson J.P., Filipsson Nyström H., Åkerblad A.C., Kołtowska-Häggström M., Johannsson G. (2014). The relationship between glucocorticoid replacement and quality of life in 2737 hypopituitary patients. Eur. J. Endocrinol..

[51] Grossman A., Johannsson G., Quinkler M., Zelissen P. (2013). Therapy of endocrine disease: Perspectives on the management of adrenal insufficiency: Clinical insights from across Europe. Eur. J. Endocrinol..

[52] Kann P.H., Bergmann S., Stalla G.K., Dimopoulou C., Weber M.M., Pedersen B.T., Meckes-Ferber S. (2017). Gender-, age- and time-dependent dosing of growth hormone in adults—real-world data from a decade of clinical practice in Germany. Gynecol. Endocrinol..

[53] van Varsseveld N.C., van Bunderen C.C., Franken A.A., Koppeschaar H.P., van der Lely A.J., Drent M.L. (2015). Tumor recurrence or regrowth in adults with nonfunctioning pituitary adenomas using GH replacement therapy. J. Clin. Endocrinol. Metab..

[54] Allen D.B., Backeljauw P., Bidlingmaier M., Biller B.M., Boguszewski M., Burman P., Butler G., Chihara K., Christiansen J., Cianfarani S. (2016). GH safety workshop position paper: A critical appraisal of recombinant human GH therapy in children and adults. Eur. J. Endocrinol..

[55] Cianfarani S. (2019). Risk of cancer in patients treated with recombinant human growth hormone in childhood. Ann. Pediatr. Endocrinol. Metab..

[56] Hindmarsh P.C., Brook C.G. (1999). Compliance with growth hormone treatment—Is it a problem?. Horm. Res..

[57] Loche S., Salerno M., Garofalo P., Cardinale G.M., Licenziati M.R., Citro G., Caruso Nicoletti M., Cappa M., Longobardi S., Maghnie M. (2016). Adherence in children with growth hormone deficiency treated with r-hGH and the easypod™ device. J. Endocrinol. Invest..

[58] Lal R.A., Hoffman A.R. (2018). Long-Acting Growth Hormone Preparations in the Treatment of Children. Pediatr. Endocrinol. Rev..

[59] Johannsson G., Feldt-Rasmussen U., Håkonsson I.H., Biering H., Rodien P., Tahara S., Toogood A., Rasmussen M.H., REAL 2 Study Group (2018). Safety and convenience of once-weekly somapacitan in adult GH deficiency: A 26-week randomized, controlled trial. Eur. J. Endocrinol..

[60] Strasburger C.J., Vanuga P., Payer J., Pfeifer M., Popovic V., Bajnok L., Góth M., Olšovská V., Trejbalová L., Vadasz J. (2017). MOD-4023, a long-acting carboxy-terminal peptide-modified human growth hormone: Results of a Phase 2 study in growth hormone-deficient adults. Eur. J. Endocrinol..

[61] Zelinska N., Iotova V., Skorodok J., Malievsky O., Peterkova V., Samsonova L., Rosenfeld R.G., Zadik Z., Jaron-Mendelson M., Koren R. (2017). Long-Acting C-Terminal Peptide-Modified hGH (MOD-4023): Results of a Safety and Dose-Finding Study in GHD Children. J. Clin. Endocrinol. Metab..

[62] Chatelain P., Malievskiy O., Radziuk K., Senatorova G., Abdou M.O., Vlachopapadopoulou E., Skorodok Y., Peterkova V., Leff J.A., Beckert M. (2017). A Randomized Phase 2 Study of Long-Acting TransCon GH vs Daily GH in Childhood GH Deficiency. J. Clin. Endocrinol. Metab..

[63] Luo X., Hou L., Liang L., Dong G., Shen S., Zhao Z., Gong C.X., Li Y., Du M.L., Su Z. (2017). Long-acting PEGylated recombinant human growth hormone (Jintrolong) for children with growth hormone deficiency: PhaseII and phase III multicenter, randomized studies. Eur. J. Endocrinol..

[64] Yang Y., Bai X., Yuan X., Zhang Y., Chen S., Yang H., Du H., Zhu H., Pan H. (2019). Efficacy and safety of long-acting growth hormone in children with short stature: A systematic review and meta-analysis. Endocrine.

[65] Harle L., Basaria S., Dobs A.S. (2005). Nebido: A long-acting injectable testosterone for the treatment of male hypogonadism. Expert Opin. Pharmacother..

[66] Zitzmann M., Nieschlag E. (2000). Hormone substitution in male hypogonadism. Mol. Cell. Endocrinol..

[67] Sykiotis G.P., Hoang X.H., Avbelj M., Hayes F.J., Thambundit A., Dwyer A., Au M., Plummer L., Crowley W.F., Pitteloud N. (2010). Congenital idiopathic hypogonadotropic hypogonadism: Evidence of defects in the hypothalamus, pituitary, and testes. J. Clin. Endocrinol. Metab..

[68] Zheng J., Mao J., Xu H., Wang X., Huang B., Liu Z., Cui M., Xiong S., Ma W., Min L. (2017). Pulsatile GnRH Therapy May Restore Hypothalamus-Pituitary-Testis Axis Function in Patients with Congenital Combined Pituitary Hormone Deficiency: A Prospective, Self-Controlled Trial. J. Clin. Endocrinol. Metab..

[69] Pitteloud N., Hayes F.J., Dwyer A., Boepple P.A., Lee H., Crowley W.F. (2002). Predictors of outcome of longterm GnRH therapy in men with idiopathic hypogonadotropic hypogonadism. J. Clin. Endocrinol. Metab..

[70] Snyder P.J., Cooper D.S., Martin K.A. Treatment of hypopituitarism. UpToDate.

[71] Olivius C., Landin-Wilhelmsen K., Olsson D.S., Johannsson G., Tivesten Å. (2018). Prevalence and treatment of central hypogonadism and hypoandrogenism in women with hypopituitarism. Pituitary.

[72] Achilli C., Pundir J., Ramanathan P., Sabatini L., Hamoda H., Panay N. (2017). Efficacy and safety of transdermal testosterone in postmenopausal women with hypoactive sexual desire disorder: A systematic review and meta-analysis. Fertil. Steril..

[73] Arlt W., Callies F., van Vlijmen J.C., Koehler I., Reincke M., Bidlingmaier M., Huebler D., Oettel M., Ernst M., Schulte H.M. (1999). Dehydroepiandrosterone replacement in women with adrenal insufficiency. N. Engl. J. Med..

[74] Johannsson G., Burman P., Wirén L., Engström B.E., Nilsson A.G., Ottosson M., Jonsson B., Bengtsson B.A., Karlsson F.A. (2002). Low dose dehydroepiandrosterone affects behavior in hypopituitary androgen-deficient women: A placebo-controlled trial. J. Clin. Endocrinol. Metab..

[75] Alkatib A.A., Cosma M., Elamin M.B., Erickson D., Swiglo B.A., Erwin P.J., Montori V.M. (2009). A systematic review and meta-analysis of randomized placebo-controlled trials in DHEA treatment effects on quality of life in women with adrenal insufficiency. J. Clin. Endocrinol. Metab..

[76] Brooke A.M., Kalingag L.A., Miraki-Moud F., Camacho-Hübner C., Maher K.T., Walker D.M., Hinson J.P., Monson J.P. (2006). Dehydroepiandrosterone (DHEA) replacement reduces growth hormone (GH) dose requirement in female hypopituitary patients on GH replacement. Clin. Endocrinol. (Oxf).

[77] Reisch N., Arlt W. (2009). Fine tuning for quality of life: 21st century approach to treatment of Addison’s disease. Endocrinol. Metab. Clin. North. Am..

[78] Hall R., Manski-Nankervis J., Goni N., Davies M.C., Conway G.S. (2006). Fertility outcomes in women with hypopituitarism. Clin. Endocrinol. (Oxf).

[79] Correa F.A., Bianchi P.H.M., Franca M.M., Otto A.P., Rodrigues R.J.M., Ejzenberg D., Serafini P.C., Baracat E.C., Francisco R.P.V., Brito V.N. (2017). Successful Pregnancies After Adequate Hormonal Replacement in Patients With Combined Pituitary Hormone Deficiencies. J. Endocr. Soc..

[80] Jonklaas J., Bianco A.C., Bauer A.J., Burman K.D., Cappola A.R., Celi F.S., Cooper D.S., Kim B.W., Peeters R.P., Rosenthal M.S. (2014). Guidelines for the treatment of hypothyroidism: Prepared by the american thyroid association task force on thyroid hormone replacement. Thyroid.

[81] Slawik M., Klawitter B., Meiser E., Schories M., Zwermann O., Borm K., Peper M., Lubrich B., Hug M.J., Nauck M. (2007). Thyroid hormone replacement for central hypothyroidism: A randomized controlled trial comparing two doses of thyroxine (T4) with a combination of T4 and triiodothyronine. J. Clin. Endocrinol. Metab..

[82] Powe C.E., Allen M., Puopolo K.M., Merewood A., Worden S., Johnson L.C., Fleischman A., Welt C.K. (2010). Recombinant human prolactin for the treatment of lactation insufficiency. Clin. Endocrinol. (Oxf).

[83] Powe C.E., Puopolo K.M., Newburg D.S., Lönnerdal B., Chen C., Allen M., Merewood A., Worden S., Welt C.K. (2011). Effects of recombinant human prolactin on breast milk composition. Pediatrics.

[84] Fenske W., Refardt J., Chifu I., Schnyder I., Winzeler B., Drummond J., Ribeiro-Oliveira A., Drescher T., Bilz S., Vogt D.R. (2018). A Copeptin-Based Approach in the Diagnosis of Diabetes Insipidus. N. Engl. J. Med..

[85] Behan L.A., Sherlock M., Moyles P., Renshaw O., Thompson C.J., Orr C., Holte K., Salehmohamed M.R., Glynn N., Tormey W. (2015). Abnormal plasma sodium concentrations in patients treated with desmopressin for cranial diabetes insipidus: Results of a long-term retrospective study. Eur. J. Endocrinol..

[86] Baldeweg S.E., Ball S., Brooke A., Gleeson H.K., Levy M.J., Prentice M., Wass J., Society for Endocrinology Clinical Committee (2018). Society For Endocrinology Clinical Guidance:Inpatient management of cranial diabetes insipidus. Endocr. Connect..

[87] Alexopoulou O., Beguin C., de Nayer P., Maiter D. (2004). Clinical and hormonal characteristics of central hypothyroidism at diagnosis and during follow-up in adult patients. Eur. J. Endocrinol..

[88] Lebbe M., Arlt W. (2013). What is the best diagnostic and therapeutic management strategy for an Addison patient during pregnancy?. Clin. Endocrinol. (Oxf).

[89] Durr J.A., Lindheimer M.D. (1996). Diagnosis and management of diabetes insipidus during pregnancy. Endocr. Pract..

[90] Ball S.G. (2007). Vasopressin and disorders of water balance: The physiology and pathophysiology of vasopressin. Ann. Clin. Biochem..

[91] Rajasekaran S., Vanderpump M., Baldeweg S., Drake W., Reddy N., Lanyon M., Markey A., Plant G., Powell M., Sinha S. (2011). UK guidelines for the management of pituitary apoplexy. Clin. Endocrinol. (Oxf).

[92] Sibal L., Ball S.G., Connolly V., James R.A., Kane P., Kelly W.F., Kendall-Taylor P., Mathias D., Perros P., Quinton R. (2004). Pituitary apoplexy: A review of clinical presentation, management and outcome in 45 cases. Pituitary.

[93] Gruber A., Clayton J., Kumar S., Robertson I., Howlett T.A., Mansell P. (2006). Pituitary apoplexy: Retrospective review of 30 patients—Issurgical intervention always necessary?. Br. J. Neurosurg..

[94] Maccagnan P., Macedo C.L., Kayath M.J., Nogueira R.G., Abucham J. (1995). Conservative management of pituitary apoplexy: A prospective study. J. Clin. Endocrinol. Metab..

[95] Arafah B.M., Harrington J.F., Madhoun Z.T., Selman W.R. (1990). Improvement of pituitary function after surgical decompression for pituitary tumor apoplexy. J. Clin. Endocrinol. Metab..

[96] Ayuk J., McGregor E.J., Mitchell R.D., Gittoes N.J. (2004). Acute management of pituitary apoplexy–surgery or conservative management?. Clin. Endocrinol. (Oxf).

[97] Arlt W., Society for Endocrinology Clinical Committee (2016). Society for Endocrinology Endocrine Emergency Guidance: Emergency management of acute adrenal insufficiency (adrenal crisis) in adult patients. Endocr. Connect..

[98] Larkin S., Ansorge O., Feingold K.R., Anawalt B., Boyce A., Chrousos G., Dungan K., Grossman A., Hershman J.M., Kaltsas G., Koch C., Kopp P. (2017). Pathology and Pathogenesis of Pituitary Adenomas and Other Sellar Lesions. Endotext.

[99] Ravnik J., Smigoc T., Bunc G., Lanisnik B., Ksela U., Ravnik M., Velnar T. (2016). Hypophyseal metastases: A report of three cases and literature review. Neurol. Neurochir. Pol..

[100] Morita A., Meyer F.B., Laws E.R. (1998). Symptomatic pituitary metastases. J. Neurosurg..

[101] He W., Chen F., Dalm B., Kirby P.A., Greenlee J.D. (2015). Metastatic involvement of the pituitary gland: A systematic review with pooled individual patient data analysis. Pituitary.

[102] Castle-Kirszbaum M., Goldschlager T., Ho B., Wang Y.Y., King J. (2018). Twelve cases of pituitary metastasis: A case series and review of the literature. Pituitary.

[103] Venneri M.A., Hasenmajer V., Fiore D., Sbardella E., Pof R., Graziadio C., Gianfrilli D., Pivonello C., Negri M., Naro F. (2018). Circadian Rhythm of Glucocorticoid Administration Entrains Clock Genes in Immune Cells: A Dream Trial Ancillary Study. J. Clin. Endocrinol. Metab..

